# Carbon Dots with Tunable Charge as Mucus-Penetrating Gene Carriers

**DOI:** 10.3390/pharmaceutics17101330

**Published:** 2025-10-14

**Authors:** Samuel Arca, Clea Witjaksono, Françoise Pons, Luc Lebeau

**Affiliations:** Laboratoire de Chemo-Biologie Synthétique & Thérapeutique, Faculté de Pharmacie, UMR 7199 CNRS, Université de Strasbourg, 67400 Illkirch, France

**Keywords:** carbon dots, gene delivery, lung, mucus, mucopenetration, maleamic acid, tunable charge

## Abstract

**Background/Objectives:** Local delivery of gene therapy products through the airways shows great promise for the treatment of a number of serious lung diseases, but its effectiveness is hampered by the mucus layer protecting the lung epithelium in the trachea and bronchi. **Methods:** To overcome this barrier, we engineered carbon dots (CDs) with mucus penetrating properties. **Results:** The CDs were synthesized by solvothermal treatment of citric acid and branched polyethyleneimine, and functionalized with maleamic acid groups to create cationic mucoinert nanoparticles with tunable charge. We characterized their interactions with a mucus model through turbidity and transport measurements, and assessed their impact on the viscoelastic properties of the biopolymer. We then demonstrated that the carriers are effective at delivering pDNA to a variety of cell models in vitro. In particular, mucus-producing Calu-3 cells cultured at the air–liquid interface (ALI) were used as a discriminating model to evaluate intracellular delivery of the genetic cargo through a thick layer of mucus at the cell surface. **Conclusions:** The functionalization of CDs with maleamic acid groups resulted in a 1000- to 10,000-fold increase in transfection efficiency in the mucus-producing model, offering new opportunities for lung gene therapy.

## 1. Introduction

The fundamental premise of gene therapy is to introduce a specific nucleic acid sequence into a diseased cell with the objective of restoring or suppressing the expression of a gene that is the source of the disease [1]. However, nucleic acids are susceptible to rapid degradation in a biological environment, where they are targeted by enzymes, including nucleases. Furthermore, the inherent anionic nature of nucleic acids presents a significant challenge as it renders these molecules unable to pass through cell membranes, which are also negatively charged and thus oppose electrostatic repulsions. The utilization of nucleic acid as a therapeutic agent thus requires the use of a carrier that preserves its structural integrity until it reaches its target, and masks its negative charge, allowing it to cross the cell membrane. The association of nucleic acids with carriers forms nanoparticulate systems, which are internalized by cells through the endocytic route. During this process, the nanoparticles (NPs) interact closely with the membrane, resulting in their entrapment within endosomes. Then, the endosomal compartments acidify to pH 5–6 and fuse with lysosomes, which are rich in degradation enzymes. Subsequently, the content of endo-lysosomes is rapidly digested, and the residues are directed to recycling pathways or eliminated from the cells via exosomes. It is therefore crucial that therapeutic nucleic acids are able to evade the endosomes before being degraded, in order to ensure their proper processing by the cell machinery. This step, known as endosome escape, is essential for full efficacy of a therapeutic nucleic acid. In most cases, only a minute quantity (approximately 1% or less) of endocytosed material is able to escape endosomal compartments while maintaining its full integrity [2].

So far, essentially two distinct families of nucleic acid carriers have been developed by scientists. Viral carriers, also known as viral vectors, are modified viruses that have been genetically altered to be replication-deficient. They can thus serve as vehicles to transport nucleic acid into target cells while minimizing potential health risks. Despite their good efficacy, viral vectors present a number of drawbacks, including immunogenicity, insertional mutagenesis, limited payload capacity, pre-existing immunity, and manufacturing difficulties [3]. Consequently, considerable effort has been invested over the past three decades in the development of synthetic carriers, including lipid-based nanoparticles, peptides, polymers, and inorganic materials [4]. In recent years, carbon-based nanostructures have emerged as a new class of non-viral nucleic acid carriers, including carbon nanotubes, nanodiamonds, and most recently, carbon dots (CDs) [5]. All non-viral nucleic acid carriers are cationic or polycationic, and associate with genetic material through electrostatic interaction with the anionic phosphate groups in the nucleic acid backbone. This process results in the formation of nano-sized complexes, most often with a positive net charge for optimal interaction with the negatively charged cell membrane and enhanced cell uptake. If non-viral nucleic acid carriers circumvent or mitigate the risks associated with viral ones, they do have their own set of challenges, such as a lower (TE) and toxicity concerns [6].

For many diseases, local delivery of therapeutics directly to the target tissue offers a number of significant advantages including enhanced therapy effectiveness and reduced systemic exposure and side-effects (e.g., liver toxicity or renal damage). Mucosa is defined as the soft tissue that lines the body’s canals and organs in the digestive, respiratory, reproductive, and ocular systems. The accessibility of most mucous tissues makes direct, non-invasive administration of therapeutics possible, ensuring their high local concentration at the target tissue. Mucosa functions primarily as part of the body’s immune system, providing a barrier against harmful agents, such as invasive pathogens (e.g., viruses, bacteria), and irritants (e.g., abrasive particles, smoke, dust). It secretes a slippery gel-like substance called mucus, which functions to lubricate and protect the epithelium surface. Mucus is composed primarily of water (∼95%), mucins (large, complex and heavily glycosylated proteins, ∼2–5%), lipids, DNA, non-mucin proteins (including actin), and cell debris [7]. Mucins, the primary functional component of mucus, are responsible for the essential and predominant viscoelastic properties of the mucus layer. They are organized into fibers and the majority of mucin glycoproteins possess terminal cysteine-rich domains that can form disulfide bridges, as well as a sialic acid and sulfate chemical groups. The reticulation of mucins through the establishment of disulfide bridges results in the three-dimensional mesh structure of mucus. The presence of sialic acid and sulfate groups contributes to a strongly negative surface charge on the biopolymer, thereby increasing its rigidity through charge repulsions [8]. The trapping of harmful agents by mucus occurs through two primary mechanisms: filtration, in case where the particle size exceeds the mesh size of the biogel, and adhesion through the establishment of electrostatic or hydrophobic interactions between the particle and the mucin fibers. Most diseases involving mucous membranes are associated with chronic inflammation, which can result in the hypersecretion of mucus and a potential reduction in its hydration. This, in turn, creates an even more effective barrier against therapeutic interventions. Mucus lining the mucosa has thus been recognized as one of the major obstacles to be overcome for promoting efficient gene transfer [9,10,11].

Various strategies have been employed to address the critical challenge of nanomedicine delivery to mucosal tissues. These include the use of mucoadhesive or mucoinert (mucopenetrating) materials, agents that regulate mucus secretion, mucolytic agents that can disrupt the mucus barrier, and absorption enhancers [12,13,14,15,16]. For gene delivery to the lung, essentially mucopenetrating and mucolytic approaches have been described [17,18]. It is now well established that hydrophobic [19,20] or positively charged [21] particles, regardless of their size, cannot efficiently penetrate mucus. Consequently, significant effort has been dedicated to decorating transfection particles with hydrophilic and neutral polyethylene glycol (PEG) [22]. However, this approach has encountered certain limitations. Repeated administration of PEGylated NPs results in the production of anti-PEG antibodies, which extensively trap and neutralize them [23]. Cationic transfection particles have been coated with anionic polymers, forming delivery vectors that enable mucus penetration [24,25]. Another strategy used virus-like particles displaying a high density of cationic and anionic charges at their surface, with a neutral net charge. This minimizes electrostatic interactions with mucins through a rapid exchange mechanism [26,27,28,29]. With regard to mucolytic approaches, these consist of the pretreatment of the airways with mucolytic agents (*N*-acetyl cysteine, nacystelyn) [30,31,32]. Other compounds, such as proteolytic enzymes, have been used for the ability to reduce the elastic properties and dynamic viscosity of mucus by cleaving amide bonds within the amino acid sequence of mucins [33]. Similarly, recombinant human deoxyribonuclease (rhDNase) has been used to degrade neutrophil-derived DNA entangled with mucin bundles [34].

In this study, our objective was to develop engineered CDs with mucus-penetrating properties for gene delivery applications. CDs, discovered in 2004 [35], are carbon-based structures with fascinating properties. These include ultrasmall size (less than 10 nm), size- and wavelength-dependent luminescence emission, resistance to photobleaching, biocompatibility, and ease of bioconjugation. CDs have been hailed as another breakthrough carbon-based nanomaterial, following the discovery of graphene (1947), nanodiamonds (1963), fullerenes (1984), and carbon nanotubes (1991) [36]. CDs are typically synthesized from carbon-containing materials and can be produced through top-down or bottom-up approaches, using a wide range of starting materials (graphite, carbon nanotubes, small organic molecules, polymers, renewable raw materials, etc.) [37]. The properties of CDs can be imparted by regulating their microstructure. In particular, coating of the surface of the carbon core of the CDs with passivation reagents may regulate the surface state of the particles. It has been demonstrated that heteroatom-containing passivation reagents can significantly enhance the intrinsic fluorescence of CDs [38]. A wide range of organic molecules can be used for surface passivation. The literature is now replete with examples, and nitrogen-containing molecules have assumed a prominent position. In addition to enhancing the fluorescence properties, incorporating nitrogen atoms at the surface of CDs may offer the opportunity to install cationic charges on the NPs by the way of ammonium groups. This prompted scientists working on nucleic acid carriers to consider the potential of polycationic CDs to assemble with nucleic acid into nano-sized complexes, as are required for cell internalization. Since the first report on the successful use of CDs for gene delivery in 2012 [39], significant efforts have been dedicated to the field as recently reviewed [5,40,41,42].

**Scheme 1:**
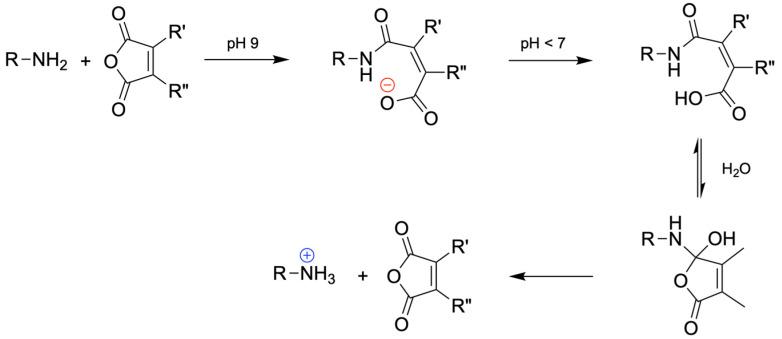
Formation and reactivity of maleamic acids (R’, R” = H or Me).

With a view of developing CDs for local delivery of gene therapy products to mucosal tissues, and possibly to the lung, we hypothesized that partial neutralization of the positive charges of the carrier could reduce electrostatic interactions between CD/DNA complexes (hereafter referred to as “dotoplexes”) and negatively charged mucus constituents. This would facilitate the diffusion of the complexes across the biopolymer towards the target epithelial cells, potentially enhancing the efficacy of the transfection process. However, this approach would also result in reduced interactions between the transfection NPs and the negatively charged cell membranes, leading to decreased cellular internalization and endosome escape of the nucleic acid cargo, which would have a detrimental impact on transfection efficiency. To circumvent this issue, we considered the reversible masking of some of the carrier’s cationic charges through amine acylation with maleic anhydrides under basic conditions (Scheme 1). These amines, which are protonated at physiological pH, and therefore carry a positive charge, are transformed into maleamic acids, which are deprotonated and negatively charged at the same pH. Amine acylation with maleic anhydride is a reversible reaction, and amide hydrolysis in the maleamic acid group is intramolecularly catalyzed by the carboxyl group under acidic conditions [43]. The decoration of cationic CDs with a controlled amount of maleamic acid units could thus provide carriers with the capability of forming dotoplexes with a reduced net positive charge, representing a potential improvement in the functionality of these carriers. The hydrolysis of the maleamic acid masking group would ensure a gradual recovery of the cationic charges over time, which should improve cell uptake. Finally, during the endosome maturation process, the pH drops to approximately 5.5, and hydrolysis of maleamide under these conditions is significantly accelerated. This should result in massive regeneration of cationic charges on the dotoplexes, enabling interaction with and destabilization of the negatively charged endosome membrane, and subsequent release of the nucleic acid cargo into the cytosol. In order to investigate this hypothesis on CDs with tunable charge and validate the scenario outlined above, we synthesized CDs decorated with various maleamic acids. The synthesis was achieved through a two-step process. First, a mixture of citric acid and branched polyethyleneimine (bPEI) was subjected to hydrothermal treatment. Then, the resulting cationic CDs were reacted with substituted maleic anhydrides. The physical and photophysical properties of the resulting CDs were determined using dynamic light scattering (DLS), and fluorescence spectroscopy. We investigated the interactions between CDs and mucus using a variety of techniques, including turbidimetry and macro-rheology. The transport of CDs through mucus was determined using a permeation assay. The capacity of the carriers to form complexes with nucleic acid was evaluated using agarose gel electrophoresis. Their ability to deliver genetic material to cells was assessed in various cell lines, with a particular focus on the mucus-producing Calu-3 cell line. The results demonstrate that CDs decorated with acid-labile maleamic acid groups exhibit enhanced transfection efficiency with minimal associated cytotoxicity, making them a promising platform for delivering genetic material to mucosal tissues.

## 2. Materials and Methods

### 2.1. Materials

Citric acid, bPEI25k (MW 25 kDa), bPEI600 (MW 600 Da), methyl maleic anhydride (MMA), dimethyl maleic anhydride (DMA), poly(acrylic acid) (PAA, MW 1800 Da), 3-(4,5-dimethylthiazol-2-yl)-2,5-diphenyl tetrazolium bromide (MTT), DNA from calf thymus (ctDNA), and type III mucin from porcine stomach (PGM) were from Merck KGaA (Darmstadt, Germany). They were used as received. Coelenterazine was from ABCR SAS (Lyon, France). Dialysis membrane (Spectra/Por 3, MWCO 1000 Da) was from Spectrum Laboratories (Rancho Dominguez, CA, USA). A549 (human lung carcinoma; CCL-185) and Calu-3 cells (human epithelial adenocarcinoma; HTB 55) were obtained from ATCC-LGC (Molsheim, France). Plasmid pCMV-GLuc (5.7 kbp) was from Nanolight Technology (Pinetop, AZ, USA), pDNA concentration refers to phosphate content. Fetal bovine serum (FBS), culture media (Dulbecco’s Modified Eagle Medium, DMEM) and supplements were from GIBCO-BRL (Cergy-Pontoise, France).

All CDs and buffer solutions were prepared with deionized (DI) water purified with an EMD Millipore Milli-Q™ integral system (resistivity ≤ 18.2 MΩ·cm, Burlington, MA, USA) and filtered through a 0.22 µm PES membrane (Millex, Merck KGaA, Darmstadt, Germany).

### 2.2. Nanoparticle Synthesis

#### 2.2.1. Synthesis of CD1

In a 50 mL borosilicate reaction flask, citric acid (2.0 g), bPEI600 (8.0 g) and water (20 mL) were combined under vigorous stirring to form a homogeneous solution. Subsequently, the mixture was heated to 220 °C in the open air to remove water (approximately 15 mL) until a viscous syrup was obtained. The reaction vessel was then fitted with a reflux condenser and the reaction mixture was stirred at this temperature for 6 h. The resulting dark brown syrup was dissolved in DI water (ca. 100 mL) and adjusted to pH 7.4 with conc. HCl. The solution obtained was transferred to a dialysis bag and allowed to equilibrate against water for 96 h, with periodic replacement of the dialysate. The resulting yellow solution in the dialysis bag was then filtered through a 0.22 µm PES membrane and freeze-dried at −50 °C for 24 h to give **CD1** (0.76 g) as a light-yellow hygroscopic powder. According to accepted nomenclature, the prepared CDs are classified as carbon polymer dots (CPDs) [44].

#### 2.2.2. Synthesis of pH-Sensitive CDs

The pH-sensitive CDs were prepared in accordance with the established protocol for the synthesis of **CD2-10**. In summary, **CD1** (50 mg) was dissolved in DI water (5 mL), and the pH was adjusted to 8–9 with 1 N NaOH. MMA (5 mg, 10% *w*/*w*) in acetonitrile (1 mL) was then added and the pH was maintained within the range of 8–9. The reaction mixture was stirred at rt overnight, after which acetonitrile was removed under vacuum. The aqueous residue was freeze-dried to yield **CD2-10**.

**CD2-20** and **CD2-30** were obtained in the same manner, with **CD1** reacting with increasing amounts of MMA (20 and 30% *w*/*w*, respectively). **CD3-10**, **CD3-20**, **CD3-30**, and **CD3-40** were prepared in a similar manner, with DMA replacing MMA (10, 20, 30, and 40% *w*/*w*, respectively).

### 2.3. Nanoparticle Characterization

TEM observations were performed on a Tecnai G2 (FEI, Hillsboro, OR, USA) microscope operating at 200 kV, equipped with a Falcon direct electron detector (Thermo Fisher Scientific, Illkirch, France). Copper grids (300 mesh) coated with a continuous carbon film were used as supports. Prior to sample deposition, the carbon film was rendered hydrophilic by a 90 s treatment in a Fischione 1070 plasma cleaner (34% power, gas mixture: argon 80%, oxygen 20%). A 5 µL drop of CD suspension (0.5 µg/µL) was deposited onto the grid and allowed to adsorb for 1 min. Then excess solution was removed, and the grid was air-dried before imaging.

The size (hydrodynamic diameter) and zeta potential (ζ) of the CDs and CD/pDNA complexes were determined by dynamic light scattering (DLS) and electrophoretic light scattering (ELS), respectively, using a Zetasizer NanoZS Pro Red apparatus (Malvern Instruments, Paris, France). The refractive index (RI) used in the equation that calculates the hydrodynamic particle radius has not been determined for these CDs. Instead, it was arbitrarily set at 1.47, which corresponds to an average value measured for N-doped CDs [45,46]. All measurements were conducted in triplicate on samples prepared in 1.5 mM NaCl, pH 7.4, at 25 °C. The data were analyzed using the multimodal intensity distribution software provided with the instrument and expressed as the mean ± SD.

The surface charge density (electrokinetic charge, Q_ek_) was determined by polyelectrolyte titration with PAA, in accordance with a previously published procedure [47]. In brief, the change in the ζ-potential of a solution of CDs (1.0 mL at 1.0 mg/mL in NaCl 1.5 mM, pH 7.4) was monitored as it was spiked with a solution of PAA (10.0 mg/mL in NaCl 1.5 mM, pH 7.4). The Q_ek_ value was calculated from the amount of PAA (mmol of acrylic acid units) required to reverse the sign of ζ (isoelectric point) as determined by interpolation of the titration curve. Q_ek_ was expressed in mmol/g.

Functionalization of CDs with MMA and DMA was assessed by FT-IR analysis using a Nicolet 380 FT-IR spectrometer in the ATR mode. Functionalization ratio was determined by ^1^H-NMR spectroscopy. In brief, ^1^H-spectrum was recorded from a mixture of **CD-1** and the grafting reagent (5.0 mg of each) in D_2_O. From ^1^H-NMR spectra obtained for the dialyzed grafted NPs, the amount of decorations (in mmol/g) were calculated by simple application of the rule of three, considering the specific NMR signal of **CD-1** (2.5–3.5 ppm), and that of the mono- or dimethyl maleamic acid moieties (1.7–2.0 ppm). ^1^H-NMR spectra were recorded on a Bruker 400 MHz Avance III instrument (Billerica, MA, USA), with chemical shifts δ reported in ppm relative to an internal reference (t-BuOH at 1.24 ppm).

The optical and photoluminescence properties of CDs were determined by recording UV-visible and fluorescence spectra of CD preparations (pH 7.4), using a multimode reader (Varioskan Lux, Thermo Fisher Scientific, Illkirch, France).

### 2.4. Preparation of CD/pDNA Complexes

The CD/pDNA complexes were prepared at varying weight ratios by gently combining equal volumes of CDs and pDNA solutions (prepared at the appropriate concentration in ultrapure water). The dotoplexes were then left to form for 30 min at rt without manipulation. Finally, the mixture was homogenized by pipetting up and down and then utilized for the various assays described below.

### 2.5. pDNA Complexation Assay

The complexation of pDNA by CDs was investigated using a nucleic acid retardation assay. To this end, freshly prepared dotoplexes (20 µL, containing 0.8 µg pDNA) were mixed with loading buffer (2 µL, Invitrogen, Waltham, MA, USA), and the resulting mixtures were loaded into the wells of a 1% agarose gel prepared in TAE buffer (40 mM Tris base, 20 mM acetic acid, 1 mM EDTA). A control well was loaded with “naked” pDNA (0.8 μg in 20 µL) supplemented with loading buffer (2 µL). The gel was then subjected to a 100 V electric field in TAE buffer for 60 min. DNA staining was performed by immersing the gel in a BET solution (0.5 µg/mL, QBiogene, Carlsbad, CA, USA) for 15 min. It was subsequently washed in a bath of ultrapure water and imaged under UV light using a gel imager (Amersham Imager, GE Healthcare, Chicago, IL, USA).

### 2.6. Turbidimetric Measurements

The interaction between CDs or the dotoplexes made thereof and the mucin fibers was examined by turbidimetry [48]. For this purpose, a solution of PGM (40 mg/mL, 50 µL) in PBS at pH 7.4 was mixed with an aqueous solution of CDs (10 mg/mL, 200 µL) or dotoplexes (200 µL, obtained after complexation of the same amount of CDs with ctDNA) in ultrapure water. Solutions containing only PBS, PGM, or CDs were prepared in parallel. Subsequently, samples (100 µL) were transferred to a 96-well plate and subjected to gentle orbital shaking at 37 °C for 1 h. At the end of the incubation period, the light-scattering ability of the solutions (λ = 600 nm) was quantified using the Varioskan Lux instrument. The experiments were conducted in triplicate, and the results were expressed as relative absorbance (ΔA) according to the following equation:

ΔA = A*_S_* − A*_CD_* − A*_PGM_* − A*_PBS_*

where A*_S_*, A*_CD_*, A*_PGM_*, and A*_PBS_* stand for the absorbances measured with the sample, the solution of CDs or dotoplexes alone, the solution of PGM alone, and PBS alone, respectively.

### 2.7. Mucopenetration Measurements

The capacity of CDs and dotoplexes to penetrate an artificial mucus layer was examined using 24-well cell culture inserts. These experiments were performed in a humidified chamber to avoid drying effects that could overlap with actual transport phenomena. In brief, a mucus layer was created by uniformly depositing 25 µL of a PGM solution (30 mg/mL in PBS) on the membrane of an insert (Falcon^®^: PET membrane, surface area of 0.33 cm^2^, pore size of 0.4 µm). The semipermeable membrane allows small molecules to pass through while retaining the mucin hydrogel in the insert. The mucus layer formed was approximately 750 µm thick, based on calculations (25 µL/0.33 cm^2^). The insert was then placed in the well of a 24-well culture plate containing 700 µL of PBS. In this configuration, the insert served as the donor compartment while the well of the plate acted as the acceptor compartment. Following a 30 min incubation at 37 °C to allow the mucus layer to reach equilibrium, a solution of CDs (12.5 µL at 10 mg/mL in DI water) or of dotoplexes prepared with ctDNA (12.5 µL at 10 mg/mL in DI water) was carefully applied to the mucus layer. Concurrently, an identical volume of the CD or dotoplex solution to be evaluated was applied to an insert containing PBS (25 µL) in lieu of mucus, serving as the control condition. After incubation at 37 °C for 5 h, the contents of each basolateral compartment were collected (700 µL) and transferred to an incubator set at 60 °C overnight to obtain dry pellets. The pellets were resuspended in DI water (110 µL), and the quantity of CDs or dotoplexes present in the resulting solutions was determined by measuring the fluorescence intensity of the samples using excitation and emission wavelengths of 370 and 460 nm, respectively, with the Varioskan Lux instrument. The translocation efficiency of the NPs through the mucus layer was expressed as a percentage, by dividing the fluorescence intensity measured for each insert with mucus by the fluorescence intensity measured for the corresponding insert without mucus (control).

### 2.8. Rheological Measurements

Oscillatory shear rheological analysis was conducted using a Haake Mars 60 rheometer fitted with a 25 mm diameter parallel-plate geometry (Thermo Electron, Karlsruhe, Germany). The viscoelastic characteristics of mucin hydrogels were evaluated both with and without CDs. Control mucin samples (100 mg/mL) were prepared by dissolving PGM lyophilizate in phosphate-buffered saline (PBS), with pH adjustment to 7.4. For CD-containing samples, PGM lyophilizate (100 mg) was dissolved in 1 mL of ultrapure water containing CDs (5 mg/mL). All mixtures underwent continuous rotational stirring at rt until achieving complete dissolution and homogeneity, followed by pH adjustment to 7.4. The rheometer plates were pre-equilibrated at 20 °C for 2 minutes before depositing 180 µL of sample. After a 2-minute equilibration period, oscillatory measurements were performed at 20 °C with a 3 mm gap between plates. The linear viscoelastic (LVE) range for each sample was established through strain amplitude sweeps (γ: 0.0001 to 10) at 1 Hz frequency. This critical step identified the appropriate strain amplitude for subsequent elastic modulus (G′) and loss modulus (G″) measurements, ensuring gentle stress application that preserves gel microstructure without disrupting cross-links and entanglements. The frequency-dependent behavior of G′ and G″ moduli was determined by measuring sample response to oscillating angular displacement. All frequency sweep measurements were conducted at a strain amplitude of 0.05 across a frequency range of 0.0178 to 1.78 Hz.

### 2.9. Cell Culture

A549 and Calu-3 lung epithelial cells were cultured in complete DMEM/F12 culture medium, i.e., containing 10% FBS, 100 U/mL penicillin, 100 μg/mL streptomycin, 5 mM HEPES and 2 mM L-glutamine. The cultures were conducted in 75 cm^2^ culture flasks at 37 °C in a humidified chamber with 5% CO_2_. At 80–100% confluency, cells were detached from the flasks by trypsin treatment (0.05% trypsin-EDTA in PBS). They were then either transferred to new flasks to maintain the culture or seeded in 96-well plates and/or 24-well inserts for experiments. A459 and Calu-3 cells were seeded in 96-well plates at a density of 6000 and 10,000 cells/well, respectively, and transfection experiments were conducted 24 h later. Calu-3 cells were seeded on inserts at a density of 100,000 cells/insert. After a period of 3 days, culture medium was removed from insert apical chamber, and the cells were further cultured at the air–liquid interface (ALI) for 7 days. During this period, the culture medium in the basolateral compartment of the inserts was replaced every 2–3 days. Transfection experiments were started on day 7 of ALI culture.

### 2.10. Transfection Assay

In cultures carried out on plates, freshly prepared dotoplexes (10 µL containing 0.4 µg of pDNA) were deposited in each well containing 100 µL of culture medium, and the transfection efficiency was assessed 24 h later. In ALI cultures, at d 7, complete culture medium (100 µL) was added to the apical compartment of each insert, followed 1 h later by freshly prepared dotoplexes (10 µL containing 1.6 µg of pDNA). Transfection efficiency was assessed 24 or 72 h later. For the 72 h condition, the dotoplexes were removed from the inserts 24 h after deposit. To achieve this, the supernatant was removed by gentle aspiration and replaced with fresh complete culture medium. In all experiments, untreated cells served as a negative control and each condition was tested in triplicate (cultures on plates) or duplicate (ALI cultures). The plasmid DNA (pCMV-GLuc) used in these experiments encodes the *Gaussia princeps* luciferase (GLuc) protein. As GLuc is an excreted protein, transfection efficiency was assessed directly by measuring luciferase activity in the culture supernatant. To this end, a solution of coelenterazine (1.5 µM, 50 µL), a GLuc substrate, was added to an aliquot of culture supernatant (20 µL), and the resulting bioluminescence was measured over a 1 s period using the Varioskan Lux instrument. Transfection efficiency was expressed in relative luminescence units (RLU).

### 2.11. Cytotoxicity Assay

In parallel to the transfection assays, cytotoxicity of the CD/pDNA complexes was evaluated by measuring cell mitochondrial activity using the colorimetric MTT assay. At the end of the transfection period (24 or 72 h), the culture medium was removed and the cells were carefully washed with PBS. Fresh complete culture medium containing MTT (1.0 mg/mL) was added (100 µL/well) and the cells were incubated at 37 °C for 1 h. After MTT removal, DMSO (100 µL) was added to lyse the cells and solubilize the reduced MTT (formazan) generated by mitochondrial succinate dehydrogenase activity. The intensity of the MTT reduction was evaluated by measuring the absorbance of the resulting solution at 570 nm, with a correction at 690 nm. Cell viability was expressed as the percentage of absorbance of dotoplex-treated cells relative to that of untreated cells. The value for each sample is the mean ± SD of three independent determinations. The cytotoxicity of the bare CDs was determined similarly, treating A549 cells for 24 h with an escalating dose of CDs.

### 2.12. Statistical Analysis

The data were expressed as mean ± SD. The statistical significance of the difference between the groups was determined by a Student’s *t*-test, with the calculations performed using the Kaleidagraph 5.0.6 software. The data were considered as significantly different when the *p* value was less than 0.05.

## 3. Results and Discussion

### 3.1. Design, Synthesis, and Characterization of CDs

**Scheme 2:**
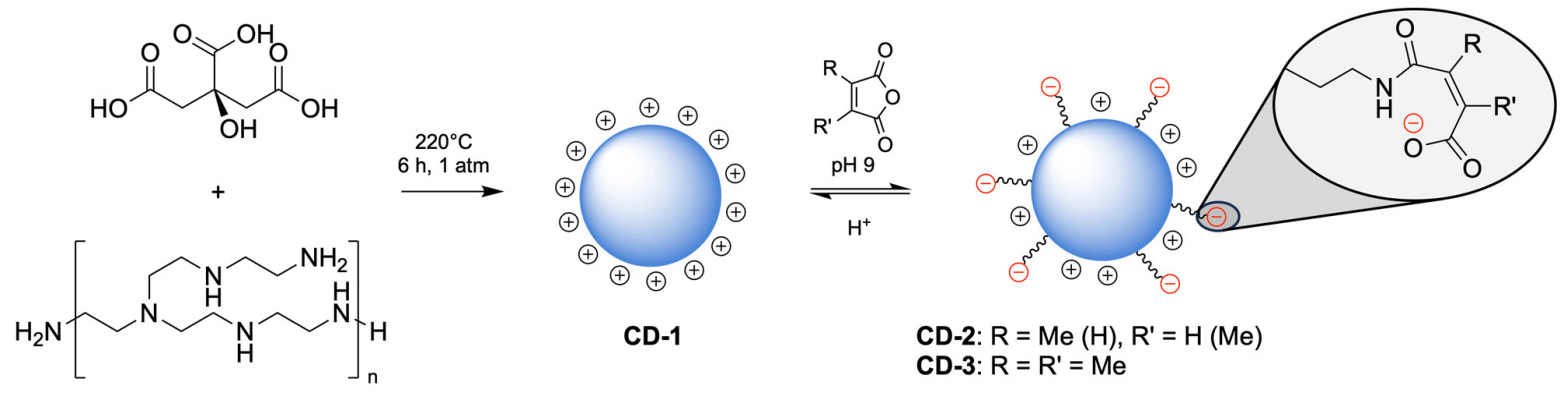
Preparation of **CD-1**, **CD-2**, and **CD-3**.

CDs that have been described to date for gene delivery applications have been exclusively prepared by a bottom-up strategy, using simple organic precursors as carbon source, including citric acid, glycerol, glucose, terephthalic acid, hyaluronic acid, folic acid, triaminobenene, or arginine [5,40,41,42]. Linear or branched PEI (lPEI, bPEI, respectively) with various molecular weights (from 600 Da to 25 kDa) or small homologous compounds (e.g., ethylenediamine, tetraethylenepentamine) have been used as passivation reagents for the installation of cationic charges on the surface of the CDs. In line with our previous reports [49,50], we selected citric acid and low molecular weight bPEI (bPEI600, MW = 600 Da) as the basis for our study. **CD1** was thus prepared by hydrothermal treatment of a mixture of citric acid and bPEI600 (1/4, *w*/*w*) for 6 h at 220 °C (Scheme 2). The resulting NPs were purified by extensive dialysis before being reacted with MMA or DMA. The reactions were conducted in water, under alkaline conditions (pH 9). The cationic net charge of the resulting CDs was modulated by using various amounts of anhydride. The reaction of **CD1** with MMA resulted in the production of **CD2-10**, **CD2-20**, and **CD2-30**, depending on the amount of anhydride involved in the reaction (10, 20, and 30%, *w*/*w*, respectively). From the Q_ek_ value measured for **CD-1** (vide infra), molar equivalents of anhydride per amino group on **CD-1** were determined to be 0.22, 0.46, and 0.70, respectively. Similarly, **CD3-10**, **CD3-20**, **CD3-30**, and **CD3-40** were produced by reacting **CD1** with DMA (molar equivalents of anhydride per amino group on **CD-1**: 0.21, 0.42, 0.63, and 0.84, respectively). The intramolecular catalysis of amide hydrolysis in maleamic acids has been investigated by various authors [43,51,52]. In particular, Kirby and Lancaster have determined that dimethylmaleamic acids may be hydrolyzed up to some three orders of magnitude more rapidly than the monomethyl analogs. This is explained by the steric compression resulting from the accumulation of four coplanar substituents on the central double bond, which increases the energy of the ground state relative to the transition state for cyclization. It is therefore anticipated that the disparate degradation rates of the differently substituted maleamide motifs will productively modulate the rate of regeneration of positive charges on CDs, thereby enabling them to better diffuse through the mucus, and escape more efficiently from the acidic endosome.

**Figure 1:**
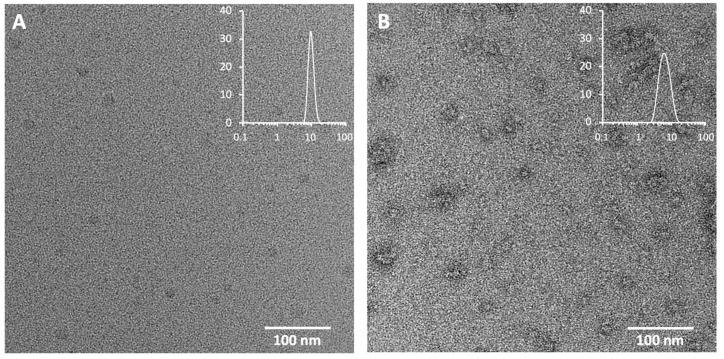
Transmission electron micrograph of **CD1** (**A**) and **CD3-30** (**B**). **CD3-30** offered poorly contrasted TEM images, and treatment of the sample with 1% uranyl formate was necessary for the visualization of the NPs. Inset: size distribution of the NPs as determined by DLS (x axis: particle size in nm; y axis: particle number in %).

The functionalization ratio (FR) of the NPs with the maleamic acid moieties was determined by NMR spectroscopy (Table 1). The results indicated that saturation was not reached and that the FR was roughly proportional to the amount of anhydride involved in the reaction. The presence of the surface functional groups on the CDs was investigated by FT-IR spectroscopy. In the FT-IR spectra of **CD1**, the broad peak around 3360 cm^−1^ and the wide absorption at 500–800 cm^−1^ were attributed to the stretching vibration and the out-of-plane vibration of N-H and O-H, respectively (Figure S1). The typical stretching vibrations of the C=O and C=N bonds were recorded at 1560 and 1625 cm^−1^. The introduction of an increasing amount of monomethyl maleamic acid decoration on the CDs (**CD2-10**, **CD2-20**, and **CD2-30**) resulted in the broadening of the peak at 1625 cm^−1^, which subsequently formed an increasingly prominent shoulder. This phenomenon was attributed to the stretching vibration of C=O in the amide groups, thereby revealing the maleamic acid decoration. This interpretation is supported by the emergence of a novel band at 1400 cm^−1^, which was ascribed to the stretching vibration of C-N in amides. In the **CD3** series, these two new bands were clearly more visible and appeared at 1700 and 1407 cm^−1^, respectively. The morphology and structure of the CDs have been characterized by transmission electron microscopy (TEM). Images demonstrate that **CD1** were uniformly dispersed globular NPs with a narrow size distribution and an average diameter of 11–14 nm (Figure 1A). Maleamic acid-decorated CDs appeared as small aggregates on the TEM grid, as exemplified with **CD3-30** (Figure 1B). Nevertheless, individual nanoparticles could be visualized, suggesting that agglomeration was artefactual and occurred during the sample preparation process (drying-induced agglomeration). While the hydrodynamic diameter of parent **CD1** was established at ca. 10 nm by DLS, decoration with maleamic acid groups resulted in a decrease in the size of the NPs, ranging from 3.9 to 6.1 nm. This can be rationalized considering weaker repulsions between adjacent PEI strands due to charge neutralization by maleamic acid residues, thus adopting a more compact arrangement around the carbon core. The zeta-potential (ζ) of parent **CD1** was +22.4 ± 0.6 mV. As anticipated, the surface charge of the grafted CDs decreased in line with the rise in maleamic acid density, thus reaching +2.6 ± 0.5 mV and +7.0 ± 0.2 mV for **CD2-30** and **CD3-40**, respectively. Determination of Q_ek_ value by polyelectrolyte titration experiments provide valuable insights into the three-dimensional charge distribution within the NPs (not solely focused on the charges displayed at the slipping plane) [44], by offering a more comprehensive assessment of the cationic charges available for complexation with anions (e.g., DNA). The Q_ek_ value of parent **CD1** was 3.8 mmol/g. As the degree of functionalization of the CDs by maleamic acids increased, the Q_ek_ values naturally decreased. The decrease in the Q_ek_ values was more rapid in the **CD2** series than in the **CD3** series. This may potentially indicate a higher reactivity of MMA with CDs when compared to DMA, which is consistent with the functionalization ratios. It may also account for some amide hydrolysis in dimethyl maleamic acid groups at pH 7.4 during the Q_ek_ value determination. In any case, the reaction of **CD1** with MMA led to a faster reduction in the net positive charge of the NPs than with DMA. All the CDs exhibited similar photophysical properties, indicating that functionalization did not change this characteristic (for full spectra, see Figure S2).

### 3.2. Nanoparticle–Mucus Interactions

**Figure 2:**
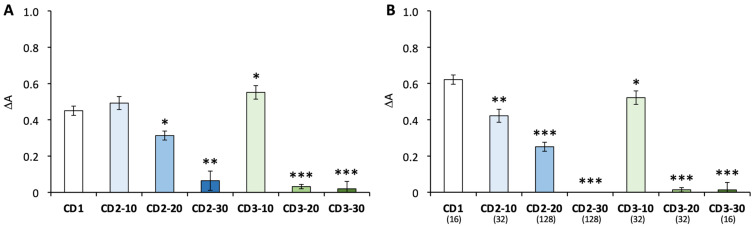
Evaluation of mucin–CD (**A**) and mucin–dotoplex (**B**) interactions by turbidimetric measurement. The CD/pDNA *w*/*w* ratios in dotoplexes are indicated into brackets. The results are expressed as the change in absorbance (ΔA) after mixing a PGM solution with CDs or the corresponding dotoplexes for 1 h at 37 °C. The results are the mean ± SD of *n* = 3 experiments. The statistical differences when compared to **CD1** were determined by Student’s *t*-test: *p* < 0.05 (*), *p* < 0.01 (**), and *p* < 0.001 (***).

It is essential to evaluate the interactions between particles and mucus components to define the capacity of these particles to diffuse through and traverse the mucus layer. It is generally accepted that low interactions are crucial for an effective mucus-penetrating nanoparticulate system. To evaluate the interactions between mucus and maleamic acid-decorated CDs or dotoplexes made thereof, we utilized porcine gastric mucus (PGM) as a simplified mucus model. We conducted turbidimetric measurements to assess the aggregation of mucin upon contact with the NPs. The aggregation of mucin results in an increase in the turbidity of the medium, which is reflected in an increase in the absorbance of the samples (ΔA). Consequently, the absorbance of a mucin solution was monitored at 600 nm upon the addition of NPs, and the resulting change in absorbance was calculated. The addition of CDs to the PGM solution resulted in a notable increase in turbidity, indicating CD–mucin electrostatic interactions and the formation of large light scattering aggregates (Figure 2A). The aggregation effect was found to diminish as the rate of maleamic acid functionalization increased, i.e., as the number of cationic charges available on the surface of the NPs decreased. It became virtually negligible at the highest functionalization ratios. The same type of experiment was conducted with dotoplexes. The CD/pDNA ratios (*w*/*w*) selected were those for which transfection efficiency of the dotoplexes was found optimum (vide infra). As anticipated, addition of dotoplexes to the PGM resulted in smaller increase in turbidity when compared to CDs, due to less interaction of the dotoplexes with the mucin. This results from the partial neutralization of the cationic charges of CDs by complexation with DNA phosphates (Figure 2B). Therefore, the turbidimetry data revealed that dotoplexes prepared from **CD2-30**, **CD3-20**, and **CD3-30** did not significantly aggregate mucins.

**Figure 3:**
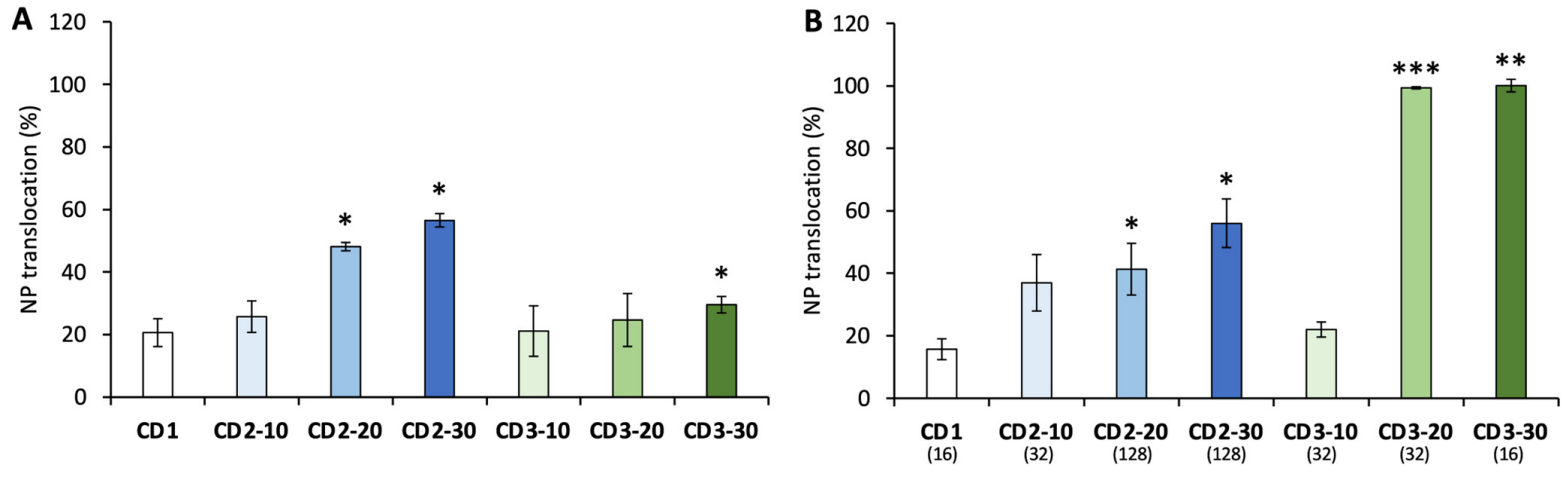
Transport of CDs (**A**) or dotoplexes (**B**) through an approximately 750 µm thick layer of mucus over a 5 h incubation period at 37 °C. CD/pDNA *w*/*w* ratios in dotoplexes are indicated into brackets. The amount of NPs that crossed the barrier is expressed as a percentage of the total amount of material deposited on the mucus layer. The results are the mean ± SD of *n* = 3–4 experiments. Statistical differences when compared to **CD1** were determined by Student’s *t*-test: *p* < 0.05 (*), *p* < 0.01 (**), and *p* < 0.001 (***).

The mucus-penetrating capacity of the various NPs was then investigated through the utilization of a translocation assay. CDs or the corresponding dotoplexes were applied to a mucin layer that had been deposited on the semi-permeable membrane of an insert. The intrinsic photoluminescence properties of CDs allowed for a straightforward assessment of their transport through the membrane-supported mucus layer, with fluorescence measurements in the basolateral compartment. The measurements were performed 5 h after the NPs were deposited onto the mucus blanket. This timeframe enabled the effective detection of CD fluorescence while allowing for the differentiation of diffusion patterns between NPs. The results demonstrate that a mere 20% of **CD1** was capable of diffusing through the mucus layer over the course of the incubation period (Figure 3A). The diffusion rate was significantly enhanced when the decoration ratio of CDs with MMA was increased. In the case of **CD2-30**, 56% was recovered in the basolateral compartment. A comparable outcome was observed with CDs modified with DMA, although the results were less discernible. The diffusion of **CD3-30** through the mucus layer was established at *ca.* 30%. With regard to dotoplexes, the MMA functionalization of CDs also led to an improvement in the diffusion of the complexes through the mucus barrier (Figure 3A). However, there was no significant difference in the translocation rate of the dotoplexes compared to the corresponding CDs. In contrast, the decoration of the NPs with DMA units had a significant impact on the diffusion rate of the subsequent dotoplexes. This resulted in the entire recovery of **CD3-20**/pDNA and **CD3-30**/pDNA complexes in the acceptor compartment. Thus, we can conclude that those dotoplexes were not significantly retained within the biopolymer matrix and were freely diffusing through it.

**Figure 4:**
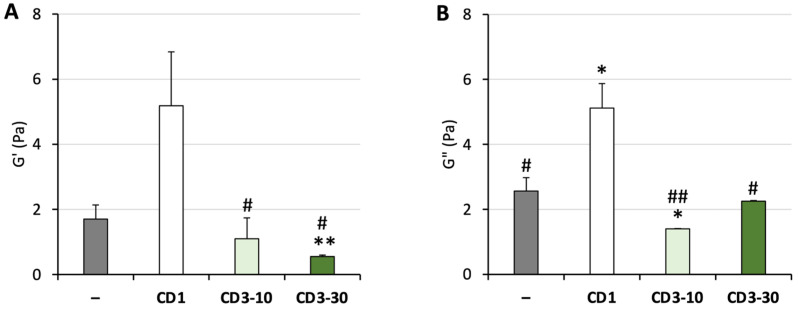
Variations in the macro-rheological elastic modulus G′ (**A**) and loss modulus G″ (**B**) of the mucus model in the presence of CDs (frequency: 1 Hz, strain amplitude: 5%). (−) corresponds to data for untreated mucus. The statistical differences when compared to untreated mucus (*) or **CD1** (#) were determined by Student’s *t*-test: *p* < 0.05 (* or #), *p* < 0.01 (** or ##).

From a macroscopic perspective, mucus can be described as a complex non-Newtonian, thixotropic gel. In response to shear stress, it exhibits characteristics of both an elastic solid and a viscous liquid and its rheological properties differ as a function of shear stress, time rate of shearing and length scale [18]. To investigate how the rheological behavior of the mucus 3D network may be affected by our positively charged NPs, we conducted macro-rheological studies and quantified variations in the viscoelastic properties of the mucin-based hydrogel provoked by CDs. The storage (G′) and loss (G″) modulus of the neat hydrogel (100 mg/mL PGM) or hydrogel mixed with **CD1**, **CD3-10**, and **CD3-30** (5 mg/mL) were determined with a strain amplitude of 5% (Figure S3). A summary of the data, focusing on G′ and G″ at a frequency of 1 Hz, revealed that **CD1** significantly enhanced the viscoelasticity of the hydrogel (Figure 4). Specifically, G′ and G″ increased from 1.70 to 5.18 Pa, and from 2.56 to 5.12 Pa, respectively. In contrast, CDs decorated with maleamic groups did not increase the viscoelasticity of mucus and even induced a slight decrease in the G′ and G″ values. These results further demonstrate the value of decorating CDs with maleamic acid units for screening interactions with mucus.

In conclusion, the qualitative results obtained from the translocation assays are consistent with those from the turbidimetric measurements and the macro-rheological studies. Specifically, our findings indicate a reduction in the interactions between mucins and CDs when they are decorated with maleamic acid groups. With regard to dotoplexes, this effect is greater with the DMA unit. However, further investigation is required to ascertain whether this is associated with the higher biodegradability of the motif under the experimental conditions when compared to the monomethylated maleamide.

### 3.3. DNA Delivery

The efficacy of CDs in complexing nucleic acid was examined by agarose gel electrophoresis. The DNA retardation assays demonstrated that all of the engineered CDs possessed the capacity to complex nucleic acid (Figure S4). The decoration of parent **CD1** with MMA units however resulted in a decline in complexation efficiency that correlates with the quantity of maleamide units installed on the surface of the NPs. Therefore, while complete pDNA complexation was achieved with **CD1** at a CD/pDNA ratio below 0.6 (*w*/*w*), this ratio increased to 0.8, 2, and was superior to 2 for **CD2-10**, **CD2-20**, and **CD2-30**, respectively. In the DMA series, however, such a pronounced effect was not observed. **CD3-10**, **CD3-20**, and **CD3-30** did fully complex pDNA at a weight ratio below 0.8. These results were consistent with the Q_ek_ values measured for the various CDs (Table 1). The electrokinetic charge Q_ek_ is a measure of the amount of cationic charges that are available for the complexation of a negatively charged polyelectrolyte at the surface of the NPs. So, the higher the Q_ek_ value, the lower the CD/pDNA ratio for achieving full plasmid complexation. This is what was observed with **CD1** for which a Q_ek_ value of 3.8 mmol/g was measured. With regard to the CD series comprising MMA decorations, a precipitous decline in the Q_ek_ value was discerned as the quantity of the maleamide units on the NPs was increased, reaching a minimum of 0.1 mmol/g for **CD2-30**. This provides insights into why full pDNA complexation could not be achieved at the higher CD/pDNA ratio evaluated in the DNA retardation assay. The decline in Q_ek_ values in conjunction with the functionalization ratio was not as pronounced in the CD series decorated with DMA units. The Q_ek_ value for **CD3-30** remained at 2.4 mmol/g, which is comparable to that of **CD2-10** (2.7 mmol/g). In conclusion, the functionalization of CDs with DMA groups has been shown to be an effective method for preserving the NPs’ ability to complex nucleic acid while minimizing interactions with mucins (vide supra).

**Figure 5:**
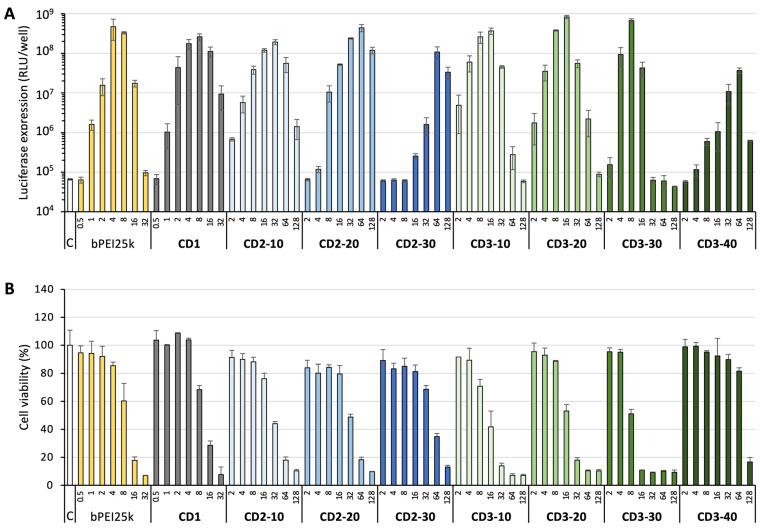
(**A**) Expression of luciferase in A549 cells treated with CD/pCMV-GLuc dotoplexes at various CD/pDNA ratios (0.5 to 128, *w*/*w*). Control (C) refers to basal bioluminescence measured in untreated cells. bPEI25k was introduced as a positive control. (**B**) Cytotoxicity of the CD/pCMV-Gluc complexes after incubation with A549 cells for 24 h, as measured by the MTT assay. Results are expressed as the percentage of the absorbance of treated cells relative to that of untreated cells. Data shown are representative of triplicate determinations (mean ± SD).

To assess the applicability of these conclusions to the intracellular gene delivery process, three in vitro models were employed to investigate the transfection efficacy of the decorated CDs. To do so, human lung carcinoma epithelial cells (A549 cell line) and human adenocarcinoma epithelial cells (Calu-3 cell line) were either conventionally cultured in 96-well plates or cultured on inserts at the air–liquid interface. The transfection efficiency of dotoplexes was evaluated by delivering a plasmid DNA expressing *Gaussia princeps* luciferase (pCMV-GLuc) into the cells. To quantify transgene expression, bioluminescence measurements were conducted in the presence of coelenterazine, a substrate for GLuc. In A549 cells, all the investigated CDs demonstrated a high transfection rate, similar to that obtained with bPEI25k, a gold standard gene delivery reagent (Figure 5A). The series of CDs modified with MMA (**CD2**) exhibited a notable improvement in transfection efficiency, demonstrating a higher efficacy than parent CDs, **CD1**. However, there was a notable decline in efficiency as the functionalization rate increased (**CD2-30** < **CD1** ≈ **CD2-10** < **CD2-20**). These findings are in line with the results of the DNA complexation assays that were presented previously. CDs modified with the DMA motif exhibited a similar pattern, with enhanced maximal efficiency. The highest transfection efficiency was observed with **CD3-20** and **CD3-30**, reaching approximately three times the level of that achieved with the parent CDs, **CD1**. This may be tentatively attributed, in whole or in part, to the accelerated hydrolysis of maleamides in the endosomal compartment during acidification. This results in an exacerbation of the electrostatic interactions between dotoplexes and the endosome membrane, which in turn leads to destabilization of the lipid bilayer and facilitates escape of the genetic material towards the cytosol. It is also possible that osmotic swelling of the endosomal compartment, following regeneration of protonatable amines on the surface of CDs, plays a role in increased endosomal escape, according to the “proton sponge” mechanism [53]. Finally, a set of hydrophobic interactions and/or steric constraints upstream of the hydrolysis step of the maleamide motif in the endosomal compartment could also contribute to the effect. For instance, higher hydrophobicity in the **CD3** series due to two methyl groups on the maleamide motif could result in enhanced hydrophobic interaction with the cell membrane, thereby promoting internalization by endocytosis. In any case, further increases in the density of MMA or DMA groups at the surface of the CDs (**CD2-30**, **CD3-40**, resp.) resulted in a decline in transgene expression, revealing optimal parameters for this approach. This phenomenon can be interpreted by considering the size of the transfection particles, as particle size influences multiple aspects of the gene delivery process from cellular uptake to intra-cellular trafficking. An increase in the size of the dotoplexes was observed with the degree of functionalization of the CDs by the maleamic acid motifs (Table S1). The hydrodynamic diameter of the dotoplexes with **CD2-30** and **CD3-40** thus established at 2277 ± 232 and 1113 ± 24 nm, resp., which is drastically higher than that with **CD2-20** et **CD3-30** (151.9 ± 1.2 and 79.0 ± 1.0 nm, resp.). This can be explained by the combined effect of a drop in the ζ-potential value and an increase in the hydrophobicity of **CD2-30** and **CD3-40** resulting from their denser decoration with the lipophilic maleamide groups, both effects favoring NPs aggregation. Though larger transfection particles generally exhibit higher transfection efficiency than smaller particles in vitro, there is an optimal size range for cell uptake that depends on the target cell type. Beyond this size, transfection efficiency decreases. Most reports in the literature indicate an optimal size ranging from 100 to 500 nm [54,55]. The size of the dotoplexes prepared with **CD2-30** and **CD3-40** definitely exceeds this range, which could explain the observed drop in transfection efficiency.

**Figure 6:**
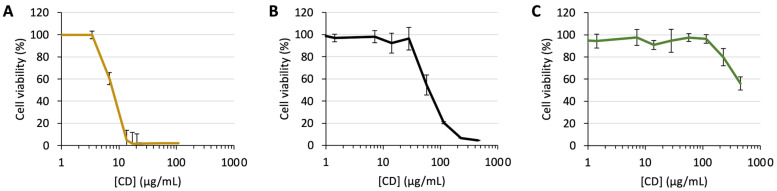
Cytotoxicity of bPEI25k (**A**), **CD1** (**B**), and **CD3-30** (**C**) in A459 cells after a 24 h incubation, as measured by the MTT assay. Basal mitochondrial activity measured in untreated cells is set at 100%. Data shown correspond to triplicate determinations (mean ± SD).

High transfection levels are typically achieved through the internalization of a significant number of transfection particles by cells via endocytosis. This process results in the accumulation of cationic charges in the intracellular milieu, which may ultimately lead to cell death by pyroptosis through the induction of lysosomal dysfunction [56]. Cell viability assessments conducted in parallel with transfection experiments were thus used to determine the safety of the dotoplexes. This was achieved using the MTT assay. In all cases, over 60–70% of mitochondrial activity was maintained at the CD concentration necessary for suboptimal gene delivery, indicating that the CDs have no or minimal adverse effect on A549 cells within the application limits (Figure 5B). To further support this conclusion, the toxicity of bare CDs has been evaluated. The cell viability was thus determined by MTT assay upon exposure of A549 cells for 24 h to an escalating dose of **CD1** or **CD3-30** (Figure 6). To provide a comprehensive view, the toxicity of the standard transfection reagent bPEI25k was also determined. The IC_50_ of **CD1** was measured at 60 µg/mL, whereas for **CD3-30**, part of whose cationic charges are balanced by the carboxylate anions of the maleamide groups, the IC_50_ was greater than 450 µg/mL. A comparison with bPEI25k, for which the IC_50_ established at 7.5 µg/mL, revealed that the bPEI-based CDs were significantly less toxic, and their decoration with the maleamic acid motif further reduced this toxicity by ca. an order of magnitude.

**Figure 7:**
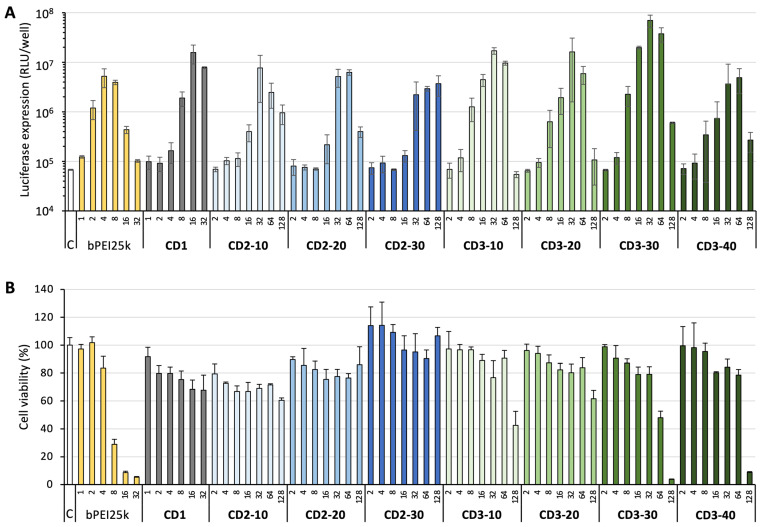
(**A**) Expression of luciferase in Calu-3 cells treated with CD/pCMV-GLuc complexes at various CD/pDNA ratios (1 to 128, *w*/*w*). Control (C) refers to basal bioluminescence measured in untreated cells. bPEI25k was introduced as a positive control. (**B**) Cytotoxicity of the CD/pCMV-Gluc dotoplexes after incubation with Calu-3 cells for 24 h, as measured by the MTT assay. The results are expressed as the percentage of the absorbance of treated cells relative to that of untreated cells. Data shown are representative of triplicate determinations (mean ± SD).

We then investigated the potential of CDs to deliver pDNA into cells covered by a mucus layer, with the aim of better simulating the conditions encountered by transfection particles when locally administered into the lung. To this end, we employed a previously developed model based on the Calu-3 cell line [57]. Calu-3 cells are mucus-producing submucosal gland carcinoma cells. Cultured at the ALI on inserts, they continuously secrete mucus. After seven days, the mucus layer thickness reaches approximately 10–12 µm, as was determined by confocal laser scanning microscopy [57]. Given the greater difficulty in transfecting Calu-3 cells compared to A549 cells, a preliminary assessment of dotoplex transfection efficacy was conducted in conventional plate cultures. As a general guideline, transgene expression in plate cultures of Calu-3 cells was approximately ten times lower than in plate cultures of A549 cells (Figure 7A). The trend observed as a function of NP decoration with maleamic acids was similar to that observed in the A549 cell line, with **CD3-30** achieving the highest transfection level while maintaining cell viability at over 80% (Figure 7B).

**Figure 8:**
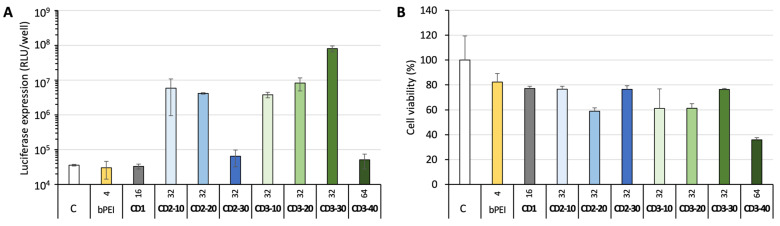
Transfection efficiency of the CD/pCMV-GLuc complexes in the Calu-3 ALI model. The CD/pDNA weight ratios used in this experiment (16 to 64, into brackets) are those found optimal for transfection of Calu-3 cells cultured in plates (cf. Figure 7). (**A**) Transgene expression was assessed 72 h after dotoplex deposit onto cells (see the body text for details). Control (C) refers to basal bioluminescence measured in untreated cells. bPEI25k was introduced as a positive control. (**B**) Cytotoxicity of the dotoplexes incubated with Calu-3 cells cultured on inserts at ALI. Cell viability was evaluated 72 h after deposit of the complexes using the MTT assay. The results are expressed as the percentage of the absorbance of treated cells relative to that of untreated cells. Data shown are representative of duplicate determinations (mean ± SD).

The CDs were then subjected to further evaluation in the Calu-3 ALI model. The mucus blanket covering cells in the ALI model efficiently trapped transfection NPs, as observed with **CD1**/pCMV-GLuc complexes (Figure 8A). The complexes were unable to induce any level of transgene expression. This must be clearly attributed to the presence of the mucus layer covering the cells, and is not a consequence of issues related to cell uptake, endosomal escape, or the timely decomplexation of the genetic material within the cells. In contrast, CDs with maleamic acid decorations furnished dotoplexes with a transfection efficiency that was almost on par with that observed in the mucus-free model. **CD3-30** demonstrated the most favorable results, performing at a level comparable to that observed in the 2D cell model, and outperforming precursor **CD-1** by up to ten thousandfold. In comparison, **CD2-10**, **CD2-20**, **CD3-10**, and **CD3-20** demonstrated efficacy levels that were approximately one to two orders of magnitude lower. These results can be benchmarked against those in the only other report available to date describing CDs specifically engineered for mucus penetration. In this report, the authors demonstrate that the decoration of CDs with PEG and various zwitterionic groups enhances CD transfection efficiency in an ALI model by a factor up to 3 [58]. It is worth noting that the highest levels of maleamic acid functionalization, whether in the mono- or dimethylated series, resulted in a drastic, if not complete, reduction in transgene expression. Finally, the decoration of CDs with maleamic acid units resulted in a superior preservation of cells in the Calu-3 ALI model relative to the mucus-free model (Figure 8B). The treatment of cells with **CD3-30**/pCMV-GLuc dotoplexes achieved the highest level of transfection while maintaining cell survival at 76% or more.

## 4. Conclusions

Cationic CDs coated with pH-labile maleamic acid functional groups were developed and their potential as gene delivery carriers has been demonstrated. Notably, the DMA decoration endowed bPEI600-based cationic CDs with remarkable mucus-penetrating properties. The transfection particles fabricated from these CDs proved highly effective for delivering pDNA to cells, with enhanced transfection efficiency attributed to a more efficient escape of the transfection particles from the endosomal compartment. Furthermore, the transfection of mucus-producing cells by maleamic acid-decorated carriers revealed highly efficient, definitely far surpassing that by vectors lacking maleamic acid groups. These findings lay the foundation for the development of new gene delivery reagents, with a particular focus on local delivery to mucosal tissues.

## Figures and Tables

**Table 1:** Physical and photophysical characteristics of CDs.

NPs	FR * (mmol/g)	Size (nm)	ζ (mV)	Q_ek_ (mmol/g)	λ_max_ (nm)	λ_ex_ (nm)	λ_em_ (nm)
**CD1**	na	10.4 ± 0.2	+22.4 ± 0.6	3.8	340	350	460
**CD2-10**	0.80	5.0 ± 0.4	+17.2 ± 4.1	2.7	345	360	460
**CD2-20**	1.34	4.5 ± 0.4	+10.2 ± 2.6	1.7	345	360	460
**CD2-30**	2.01	4.6 ± 0.3	+2.6 ± 0.5	0.1	345	360	460
**CD3-10**	0.57	3.9 ± 0.7	+18.4 ± 0.6	3.5	345	360	460
**CD3-20**	1.13	4.5 ± 0.4	+10.1 ± 0.6	3.3	345	360	460
**CD3-30**	1.91	6.1 ± 0.5	+7.5 ± 0.5	2.4	345	360	460
**CD3-40**	nd	8.1 ± 0.7	+7.0 ± 0.2	2.2	345	360	460

* FR: functionalization ratio. na: not applicable. nd: not determined.

## Data Availability

Data will be made available on request.

## Supplementary Materials

The following supporting information can be downloaded at: https://www.mdpi.com/article/10.3390/pharmaceutics17101330/s1, Figure S1: Fourier transform infrared spectroscopy (FT-IR) of the CDs; Figure S2: Photoluminescence spectra of CDs at different excitation wavelengths; Figure S3: Variations of the macro-rheological elastic modulus G′ (A) and loss modulus G″ (B) of the mucus model (PGM 100 mg/mL) in the presence of CDs (5 mg/mL) as a function of frequency at strain amplitude of 5%. “Untreated” refers to PGM sample without CDs; Figure S4: Agarose gel electrophoresis of the dotoplexes prepared from **CD1** or the different maleamide-decorated CDs, at CD/DNA *w*/*w* ratios between 0.1 and 2. In all gels, the left lane received naked DNA; Table S1: Size and ζ-potential of dotoplexes at the *w*/*w* ratio found optimal for transfection in the Calu-3 ALI model.

## Abbreviations

The following abbreviations are used in this manuscript:

ALI	air–liquid interface
bPEI	branched polyethyleneimine
CDs	carbon dots
ctDNA	calf thymus DNA
DI	deionized water
DLS	dynamic light scattering
DMA	dimethyl maleic anhydride
DMEM	Dulbecco’s modified Eagle medium
DMSO	dimethyl sulfoxide
ELS	electrophoretic light scattering
FBS	Fetal bovine serum
FR	functionalization ratio
lPEI	linear polyethyleneimine
MMA	methyl maleic anhydride
MTT	3-(4,5-dimethylthiazol-2-yl)-2,5-diphenyl tetrazolium bromide
MWCO	molecular weight cut-off
NMR	nuclear magnetic resonance
NPs	nanoparticles
PAA	poly(acrylic acid)
PBS	phosphate-buffer saline
pDNA	plasmid DNA
PEG	polyethylene glycol
PES	polyether sulfone
PGM	type III mucin from porcine stomach
ppm	parts per million
rhDNase	recombinant human deoxyribonuclease
RI	refractive index
SD	standard deviation
