# Supplementary material for: Carbon Dots with Tunable Charge as Mucus-Penetrating Gene Carriers
Source: Pharmaceutics. 2025 Oct 14;17(10):1330. doi: 10.3390/pharmaceutics17101330 (PMC12567123; doi:10.3390/pharmaceutics17101330)
Supplement: Supplementary file 1 [file pharmaceutics-17-01330-s001.zip › pharmaceutics-3865774-supplementary.pdf]

Carbon dots with tunable charge as mucus-penetrating gene carriers

Samuel Arca, Clea Witjaksono, Françoise Pons, Luc Lebeau

Supplementary information

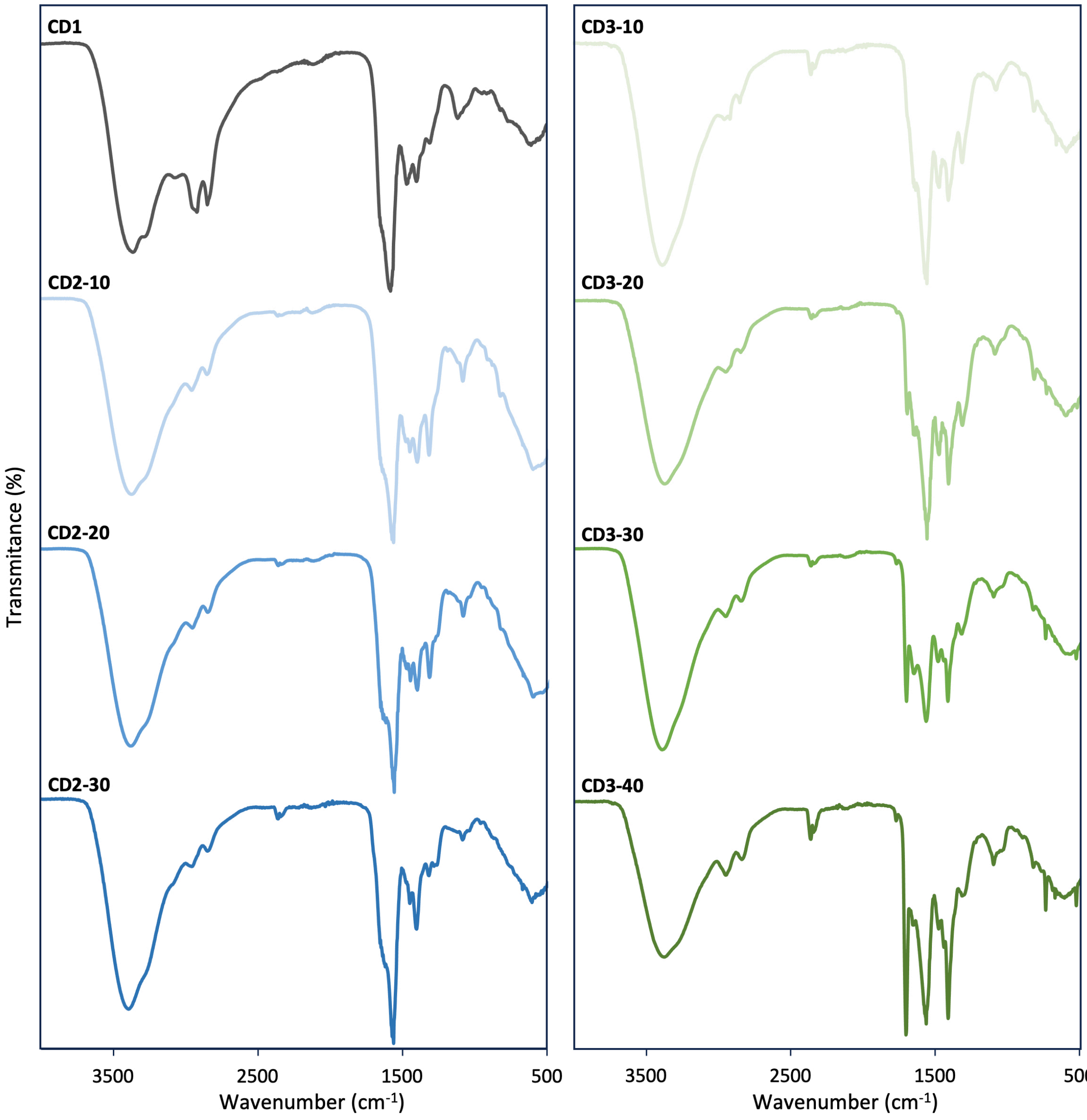

Figure S1. Fourier transform infrared spectroscopy (FT-IR) of the CDs.

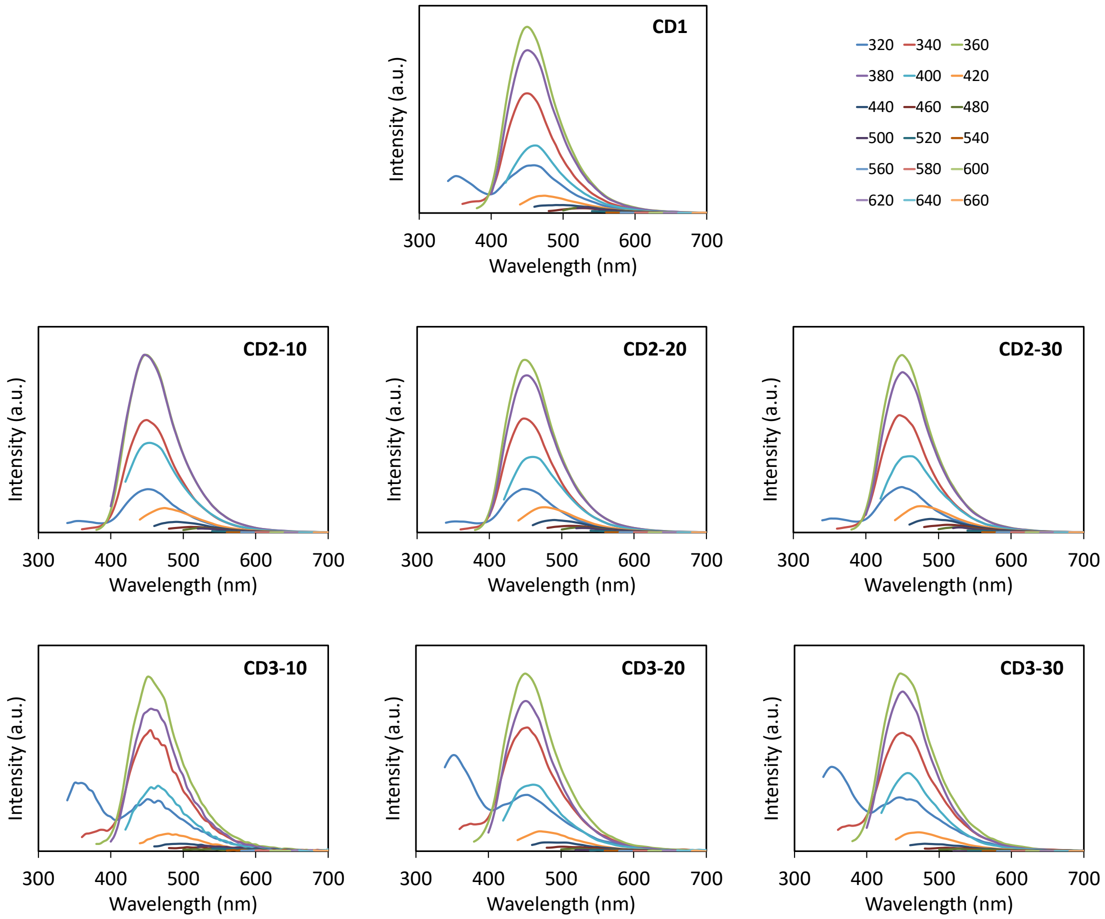

Figure S2. Photoluminescence spectra of CDs at different excitation wavelengths.

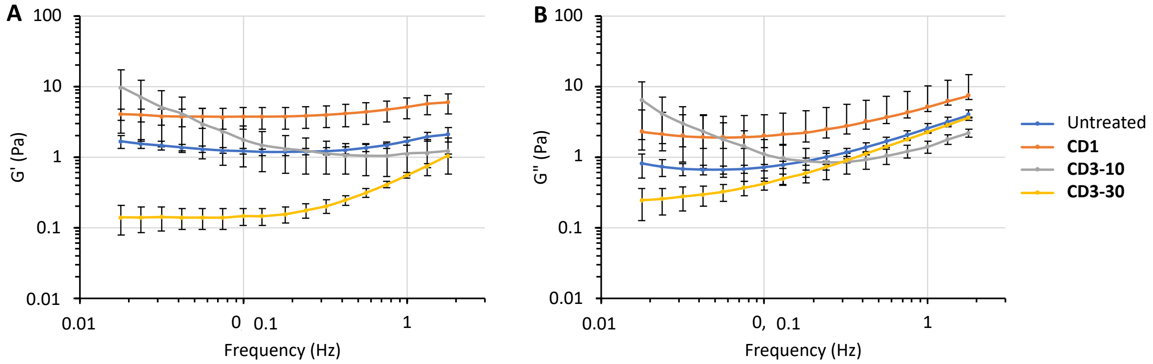

Figure S3. Variations of the macro-rheological elastic modulus G' (**A**) and loss modulus G" (**B**) of the mucus model (PGM 100 mg/mL) in the presence of CDs (5 mg/mL) as a function of frequency at strain amplitude of 5%. "Untreated" refers to PGM sample without CDs.

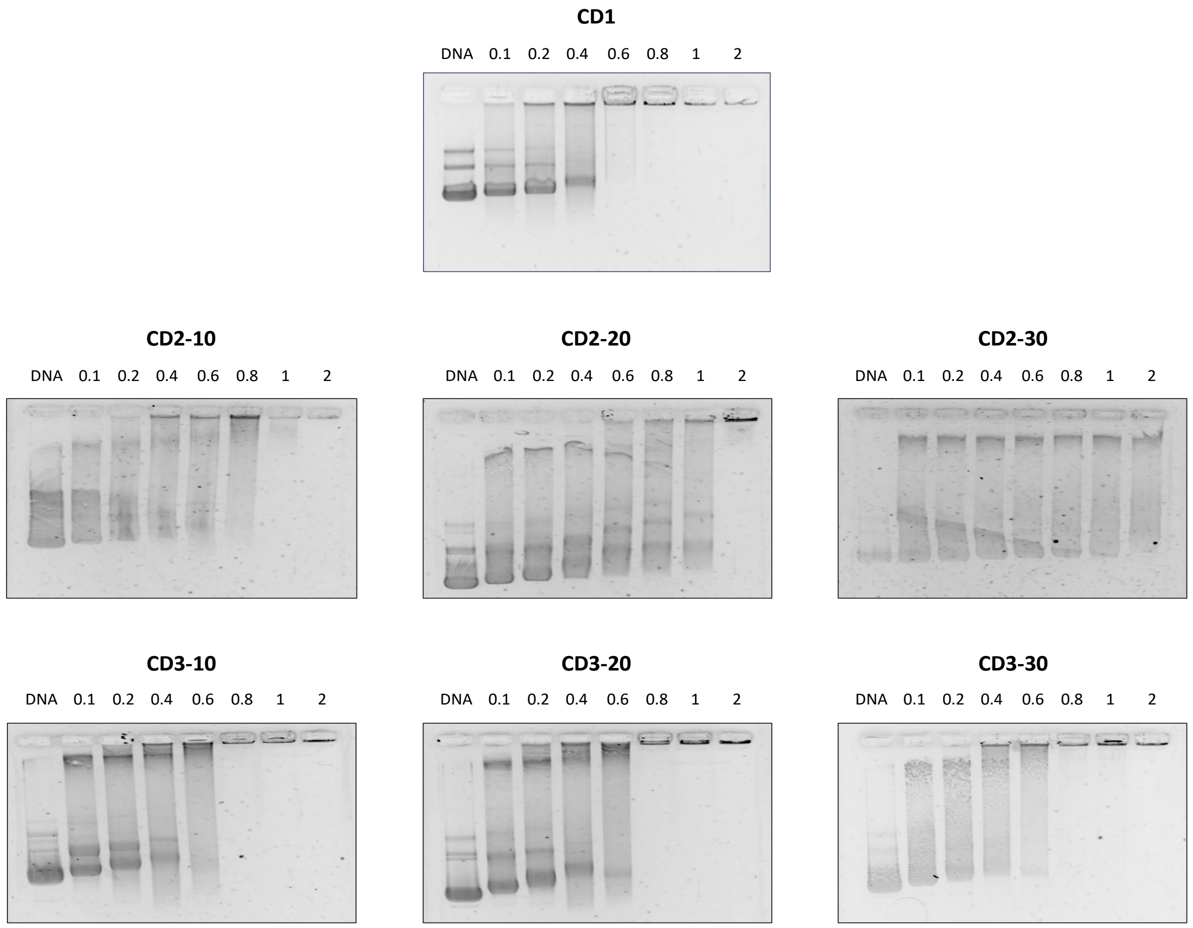

Figure S4. Agarose gel electrophoresis of the dotoplexes prepared from **CD1** or the different maleamide-decorated CDs, at CD/DNA w/w ratios between 0.1 and 2. In all gels, the left lane received naked DNA.

Table S1. Size and ζ-potential of dotoplexes at the w/w ratio found optimal for transfection in the Calu-3 ALI model.

NPs	CD/DNA (w/w)	Size (nm)	ζ (mv)
**CD1**	8	82.9 ± 0.8	+45.6 ± 0.7
**CD1**	16	55.0 ± 0.1	+49.6 ± 1.8
**CD2-10**	32	57.8 ± 0.2	+23.0 ± 0.2
**CD2-20**	32	151.9 ± 1.2	+18.2 ± 0.6
**CD2-30**	32	2277 ± 232	+5.2 ± 0.5
**CD3-10**	32	57.3 ± 0.6	+27.3 ± 0.6
**CD3-20**	32	86.1 ± 0.5	+18.2 ± 0.7
**CD3-30**	32	79.0 ± 1.0	+17.5 ± 0.8
**CD3-40**	64	1113 ± 24	+8.8 ± 0.1
